# Obesity-Associated Alterations of Natural Killer Cells and Immunosurveillance of Cancer

**DOI:** 10.3389/fimmu.2020.00245

**Published:** 2020-03-13

**Authors:** Ina Bähr, Julia Spielmann, Dagmar Quandt, Heike Kielstein

**Affiliations:** ^1^Institute of Anatomy and Cell Biology, Medical Faculty of Martin Luther University Halle-Wittenberg, Halle (Saale), Germany; ^2^Regenerative Medicine Institute (REMEDI) at CÚRAM Centre for Research in Medical Devices, School of Medicine, College of Medicine, Nursing and Health Sciences, National University of Ireland Galway, Galway, Ireland

**Keywords:** NK cells, obesity, adipokines, inflammation, cancer, prevention, immunosurveillance

## Abstract

Obesity is accompanied by a systemic chronic low-grade inflammation as well as dysfunctions of several innate and adaptive immune cells. Recent findings emphasize an impaired functionality and phenotype of natural killer (NK) cells under obese conditions. This review provides a detailed overview on research related to overweight and obesity with a particular focus on NK cells. We discuss obesity-associated alterations in subsets, distribution, phenotype, cytotoxicity, cytokine secretion, and signaling cascades of NK cells investigated *in vitro* as well as in animal and human studies. In addition, we provide recent insights into the effects of physical activity and obesity-associated nutritional factors as well as the reduction of body weight and fat mass on NK cell functions of obese individuals. Finally, we highlight the impact of impaired NK cell physiology on obesity-associated diseases, focusing on the elevated susceptibility for viral infections and increased risk for cancer development and impaired treatment response.

## Introduction

Overweight and obesity are defined as abnormal or excessive fat accumulation that is commonly classified by the body mass index (BMI), determined by dividing the body weight in kilograms by the square of the height in meters (kg/m^2^) of an individual (1). Over the past decades, the global prevalence of overweight and obesity in children and adolescents has increased dramatically in industrialized as well as in developing countries. In 2016 the World Health Organization (WHO) classified 124 million children (7%) and 650 million adults (13%) as obese worldwide (1). Overweight and obesity are commonly caused by an excess energy consumption and a decreased physical activity, but also by sleep restriction and a greater uptake of endocrine disruptors as well as social, genetic, and epigenetic factors contribute to the increasing prevalence of obesity (2–5). The escalating global epidemic of overweight and obesity is a major public health and economic problem, as excess body weight is associated with almost 4 million deaths and 120 million disability-adjusted life-years (6). In detail, overweight and obesity are significant risk factors for a number of chronic disorders, including cardiovascular diseases and type 2 diabetes mellitus as well as renal, musculoskeletal, and psychological disorders (7–10). Additionally, the susceptibility to infections and the incidence for several cancer types, like esophageal, renal, endometrial, liver, pancreatic, prostate, postmenopausal breast, and colorectal cancer, are increased in obesity (11–14). Until now, the pathophysiological processes for the increased cancer risk in obesity remain unresolved. Besides altered adipokine levels, increased secretion of growth factors and steroid hormones, oxidative stress, an altered microbiome, and low-grade inflammation have been discussed to influence cancer development in obese individuals (15–17). The excessive fat accumulation promotes a state of a systemic chronic low-grade inflammation, a condition characterized by an increased secretion of pro-inflammatory factors caused by the augmented number of adipocytes and immune cells in adipose tissue (18). Furthermore, obesity is associated with alterations in the functionality of several immune cells, like macrophages, B and T lymphocytes, mast cells, and natural killer (NK) cells (19–22).

## NK Cells on the Edge of Innate and Adaptive Immunity

NK cells are large granular lymphocytes that mediate rapid innate immunity against viruses, bacteria, parasites, and tumor cells without prior sensitization. NK cells primarily develop and mature in the bone marrow, migrate through the bloodstream, and seed into secondary lymphoid organs, like thymus, spleen, and lymph nodes as well as in other peripheral tissues, like lung, liver, kidney, uterus, and the gastrointestinal tract (23). In blood, NK cells represent 1–6% of all leukocytes and comprise up to 15% of human peripheral blood mononuclear cells (PBMCs). Human NK cells have been defined by the expression of adhesion molecule CD56 and by the absence of the T cell marker CD3. Based on their expression level of CD56 and the Fcγ receptor CD16, NK cells are subdivided into different subpopulations. The two major and classically defined subsets are the CD56^dim^CD16^bright^ NK cells and the CD56^bright^CD16^dim/neg^ NK cells. CD56^dim^CD16^bright^ NK cells represent about 90% of all NK cells and are predominantly found in peripheral blood (24). They are mostly responsible for cytotoxic activity and target cell killing but are also a source of pro-inflammatory cytokines and chemokines (25, 26). CD56^bright^CD16^dim/neg^ NK cells represent up to 10% of peripheral blood NK cells, are most frequently found in lymph nodes, and can be subdivided according to their CD16 expression into CD56^bright^CD16^dim^ NK cells and CD56^bright^CD16^neg^ NK cells (27–29). They mainly play an immunoregulatory role by abundantly producing cytokines, but can also become cytotoxic after activation, for example, by interleukins (24, 28, 30–33). Two developmental models of the two major NK cell subsets CD56^dim^ and CD56^bright^ NK cells are discussed. On the one hand, it has been proposed that CD56^bright^ CD16^dim/neg^ NK cells are precursors for CD56^dim^CD16^bright^ NK cells but can also result from activated CD56^dim^CD16^bright^, which upregulated CD56 and lost CD16 (34). On the other hand, it is discussed that both NK cell subsets have their origin in different hematopoietic lineages (28, 35).

In mice, commonly used surface markers to identify NK cells are NKp46, CD49b (DX5), and CD161 (NK1.1) gated on CD3-negative cells. In some mouse strains, such as BALB/c, NK cells are identified only by CD49b and NKp46, as these strains have allelic variants of the NK1.1 and do not react with the anti-NK1.1 antibody PK136 (36–39). In contrast to human NK cells, the surface density of CD27 and CD11b can be used to subdivide murine NK cells into four subsets that define different levels of maturation: CD11b^low^CD27^low^, CD11b^low^CD27^high^, CD11b^high^CD27^high^, and CD11b^high^CD27^low^ (40, 41). These four different subsets correspond to the stages of their maturation and were described to be associated with a progressive acquisition of NK cell functionality. Thus, CD11b^high^CD27^high^ NK cells exhibit the highest levels of both cytokine production and cytotoxicity (40, 42). Moreover, studies of Crinier et al. indicated a correspondence between the murine CD11b^+^CD27^−^ subset with the human CD56^dim^ subset and the murine CD11b^−^CD27^+^ subset with the human CD56^bright^ subset of NK cells (43).

In rats, two major NK cell subsets have been described, based on the expression of the Ly49s3 and NKR-P1B cell receptor (44, 45). Both NK cell subsets are potent in cytotoxicity and cytokine secretion, but show differences in expression of other surface NK cell receptors and proliferative capacity (46).

Recently, NK cells have been classified as one group of innate lymphoid cells (ILCs) developing from a common innate lymphoid progenitor on the bases of their transcription factor profile (47). Conventional NK cells circulate in the blood stream; however, some NK cell subsets are also known to be tissue-resident and have been identified in various tissues like uterus, skin, kidney, salivary glands, and adipose tissue (48, 49). Tissue-resident NK cells differ in the equipment of phenotyping markers, transcription factors, and cytokine secretion and may contribute therefore locally to the immunosurveillance of cancer (48–50).

NK cells mediate their cytotoxicity against target cells via two mechanisms: secretion of cytotoxic molecules or by death receptor-mediated apoptosis. NK cells can directly kill target cells by secreting a large number of cytolytic granules containing granzymes and perforins in a rapid and efficient manner. In addition, NK cell cytotoxicity can be mediated via activation of death cell receptors on target cells via binding of death ligands, e.g., FasL (Fas ligand) and TRAIL (tumor necrosis factor-related apoptosis-inducing ligand), expressed on NK cell surface, resulting in classical caspase-dependent apoptosis. Interestingly, these killing pathways are not mutually exclusive, but rather, NK cells are able to switch their strategies over the course of an immune response, and the same NK cell is able to perform serial killing (51, 52). Furthermore, NK cells are able to induce cell lysis via antibody-dependent, cell-mediated cytotoxicity, in which FcγIII receptors on NK cells recognize the Fc portion of the IgG antibody on target cells (53). Besides their cytolytic function, activated NK cells are able to produce a number of cytokines and chemokines, like TNF (tumor necrosis factor)-α, IFN (interferon)-γ, or GM-CSF (granulocyte macrophage colony-stimulating factor), in order to co-stimulate other cells of the immune system (54, 55).

The activity of NK cells is regulated by the balanced expression of activating and inhibitory surface receptors triggering a cascade of events inside the cells. In humans, the NK cell receptor repertoire essentially includes three major receptor families: human killer immunoglobulin-like receptors (KIRs) as well as natural cytotoxicity receptors (NCRs) and C-type lectin-like receptors. Most relevant activating NK cell receptors include the NCRs NKp30, NKp44 and NKp46, and the natural killer group (NKG) 2D receptor as well as DNAX accessory molecule-1 (DNAM-1) and the short-tail members of KIRs (56–58). Inhibitory NK receptors comprise the long-tail members of the KIR family, NKG2A and the killer cell lectin-like receptor subfamily G (KLRG) 1 (58, 59). Although several NK cell receptors, like NKp46 and NKG2D, are expressed in both species, the NK cell receptor repertoire in mice differs from humans. In particular, the counterpart for human KIRs is represented by the lectin-like Ly49 receptor family in mice (58). In addition, mice lack the expression of NKp44 and NKp30; however, NKp30 is an unexpressed pseudogene in mice (60, 61). Although some ligands for activating and inhibitory receptors are known today, the family of ligands is not yet fully elucidated (62).

## The Influence of Adipokines on NK Cell Functionality *in vitro* and *in vivo*

Besides the storage of energy, adipose tissue has been identified as a highly active endocrine organ secreting numerous adipokines, like leptin, adiponectin, resistin, estrogens, and interleukin (IL)-6. These adipokines do not only function locally in adipose tissue, but have very distant effects on other organs, like liver, muscle, and brain, and therefore play an important role in the regulation of energy metabolism, influence reproduction, or cardiovascular function (63). The dysregulated secretion of adipokines by an excessive growth of adipose tissue in obesity has been shown to contribute to the pathogenesis of obesity-related diseases, like type 2 diabetes, cardiovascular events, and non-alcoholic fatty liver disease (64). Adipokines also affect the functionality of several immune cells. Besides T and B lymphocytes, macrophages, mast cells, and eosinophils, NK cell physiology is influenced by hormones and cytokines secreted by adipose tissue (65).

### Studies on Leptin

Leptin is the best-characterized adipokine whose circulating plasma concentration correlates with the amount of adipose tissue and therefore is increased in obesity (66). Investigations demonstrated that the leptin receptor (obesity receptor, ObR) is expressed on splenic and peripheral blood NK cells of rats as well as on human hepatic and peripheral blood NK cells (67–76) (Table 1). Although leptin receptor expression levels on NK cells are rather dim, functional data upon leptin treatment performed by different laboratories demonstrate a functional role for this receptor on NK cells (69–73, 77).

**Table 1:** Expression of adipokine receptors on human and rodent NK cells.

**Adipokine**	**Receptor and *alternative names***	**Species**	**Detection of receptor expression in cell lines/ tissues**	**Percentage of surface receptor expression level [% of NK cells]**	**Obesity-related changes in receptor expression level**	**References**
Leptin	Leptin receptor (LEP-R) *Obesity receptor (ObR)* *CD295*	Human	NK-92 cells	Ø	Ø	(67, 68)
			YT cells	Ø	Ø	(68)
			Peripheral blood NK cells	Very dim–dim (0–9%)	ow/ob = nw	(69–73, 77)
			Hepatic NK cells	Ø	ob = nw	(74)
		Rodent (rat)	Peripheral blood NK cells	Ø	ob > nw	(75)
			Splenic NK cells	Ø	ob = nw	(75, 76)
Adiponectin	Adiponectin receptor 1/2 (AdipoR1/2) *Progestin and adipoQ* *receptor 1/2 (PAQR1/2)*	Human	NKL/NK-92/KHYG cells	Ø	Ø	(78)
			Peripheral blood NK cells	Intermediate–bright (20–90%)	ob = nw (AdipoR1); ob < nw (AdipoR2)	(70, 79, 80)
		Rodent (murine)	Splenic NK cells	Intermediate (15–17%)	Ø	(79, 81)
Interleukin-6	IL-6 receptor (IL-6R, IL-6Ra) *CD126*	Human	NK-92 cells	Ø	Ø	(82)
			Peripheral blood NK cells	Dim (5%)	Ø	(83)
			Splenic NK cells	Dim (6%)	Ø	(83)
			Tonsil NK cells	Intermediate (15%)	Ø	(83)
		Rodent (murine)	Peripheral blood NK cells	Intermediate (8–25%)	ob > nw	(84)
			Hepatic NK cells	Dim (8–12%)	ob = nw	(84)
			Perigonadal adipose tissue	Intermediate (5–15%)	ob > nw	(84)
			Splenic NK cells	Intermediate (15–40%)	Ø	(85, 86)
Estrogens	Estrogen receptor α/β (ESR1/2)	Human	Peripheral blood NK cells	Ø	Ø	(87)
		Rodent (murine)	Splenic NK cells	Bright	Ø	(88)
			Uterine natural killer cells	Bright	Ø	(89)

*AdipoR, adiponectin receptor; CD, cluster of differentiation; ESR, estrogen receptor; IL-6R, interleukin-6 receptor; NK cell, natural killer cell; nw, normal weight; ob, obese; ObR, obesity receptor (leptin receptor); ow, overweight; PAQR, progestin and adipoQ receptor; Ø, no data. Alternative receptor names are presented in italics*.

Leptin was shown to influence proliferation as well as migration of NK cells (68, 90, 91). Conflicting data exist about the impact of leptin on NK cell cytotoxicity and cytokine secretion. Several studies demonstrated that leptin increases the cytolytic activity of NK cells (67–69, 90, 92). All these studies have been performed using the human myelogenous leukemia line K652 or the murine lymphoma cell line YAC-1 as target cells for cytotoxicity assays. Interestingly, data using other target cells, like colon or breast cancer cells, revealed evidence for a decreased cytotoxicity of NK cells upon leptin treatment (67, 93). Therefore, the influence of leptin on NK cell cytolysis may depend on the tumor target cells, most likely based on their differential NK cell ligand profile. In addition to effects on NK cell cytotoxicity, leptin administration leads to conflicting results in terms of expression and secretion of cytokines, surface receptors, and enzymes as well as the cell metabolism of NK cells. On the one hand, some studies demonstrated a stimulating leptin effect on the metabolic activity, mRNA expression or release of perforin, granzyme A, IFN-γ, TRAIL, FasL, and leptin receptors of NK cells (67–69, 94). On the other hand, leptin had been shown to decrease IFN-γ secretion, mRNA expression of the activating NK cell receptors NKG2D and NKp46, and the activation marker CD69 as well as the FasL and TRAIL mRNA expression (67, 69, 93). Other studies did not find leptin-related effects on granzyme and perforin secretion nor degranulation marker CD107a, IFN-γ, or FasL mRNA expression (68, 69). These contradictions may have resulted from the usage of different leptin concentrations, incubation times, measurement of mRNA expression, or secreted proteins as well as the use of different NK cell lines or primary NK cells. Interestingly, our own studies demonstrated a leptin-induced reduction of IFN-γ secretion in primary human NK cells but not in NK-92 cells. In addition, the data point toward a decreased basal lytic activity against target cells in NK-92 cells compared to primary NK cells (93). Although NK cell lines, like NK-92, are well-established human models for investigations on NK cell functionality and exhibit phenotypical and functional characteristics of primary NK cells, these data underline the limited transferability of findings obtained using immortalized cell lines to conclude the mode of action of primary NK cells (93, 95, 96). Furthermore, results showed that the leptin effects on NK cells are more pronounced using higher leptin concentrations for incubation experiments (67, 93). These findings indicate that leptin negatively affects NK cell activity primarily in pathologically elevated leptin concentrations as determined in obese individuals.

Besides the described *in vitro* investigations of leptin on NK cells, the impact of this adipokine has also been examined *in* and *ex vivo*. The administration of leptin in wild-type rats and mice led to an increased number of NKs in blood, spleen, and liver (75, 90, 97, 98). Interestingly, elevated NK cell numbers as well as a higher NK cell killing activity after administration of pathophysiologically high leptin concentrations was solely observed in normal-weight animals, but not in obese animals (75). In addition, investigations on human primary NK cells demonstrated that treatment with high leptin concentrations increased the IFN-γ production in NK cells isolated from normal-weight individuals, but not from obese individuals (72). In contrast, leptin in physiological concentrations decreased the cytotoxicity of NK cells isolated from normal-weight but not from obese human subjects (99). These data suggest that NK cells of obese individuals are resistant to regulatory effects of leptin. Leptin mediates its cellular effects primarily via the leptin receptor-linked activation of JAKs (Janus kinases) and STATs (signal transducers and activators of transcription), which was also confirmed for NK cells (68, 100). Studies on rat and human PBMCs reported an altered post-receptor signaling in obesity, as the leptin-induced activation of JAK/STAT signaling components were diminished in obese individuals compared to normal weight subjects (69, 75). A central leptin resistance in obesity is widely accepted; therefore, a peripheral leptin resistance with an abrogated JAK/STAT signaling pathway is likely and would explain the reduced biological activity after leptin challenge in NK cells of obese individuals (69, 72, 75, 101). Furthermore, an animal study in leptin receptor deficient *db/db* mice demonstrated that the number of NK cells was decreased in blood, spleen, liver, and lung (92). In addition, the cytotoxicity as well as the expression of the activation marker CD69 of NK cells isolated from mice lacking the leptin receptor was declined compared to NK cells isolated from wild-type mice (92). These findings suggest that leptin is involved in NK cell development, activation, and function (92).

To conclude, the reported effects for leptin on NK cells are varying, as also summarized in Table 2. Therefore, several technical or biological requirements like a pure leptin preparation, an elimination of indirect effects, and the exclusion of a second receptor need to be fulfilled in order to interpret the data correctly.

**Table 2:** Effects of adipokine treatment on human and rodent natural killer cells.

**NK cell parameter**	**Leptin**		**Adiponectin**		**Interleukin-6**		**Estrogen**	
	**Human**	**Rodent**	**Human**	**Rodent**	**Human**	**Rodent**	**Human**	**Rodent**
Number	↔	↑↑ (rat) ↑↑ (murine)	Ø	↓↓ (murine)	Ø	Ø	Ø	↑ (murine)
Cytotoxicity	↓*↓↓*/↑*↑↑*	↑ (rat) ↑*↑↑* (murine)	↑/↔	↓*↓↓* (murine)	↓*↓↓↓↓*/↑*↑↑*	↓ (murine)	↓	↓*↓↓↓↓↓↓↓* (murine)
Expression of activating receptors	↓↓/↑↑/↔	↑ (murine)	Ø	↓/↑ (murine)	↑/↔	Ø	Ø	↓↑ (murine)
Expression of inhibiting receptors	Ø	Ø	Ø	Ø	↔	Ø	Ø	↑ (murine)
Migration	Ø	Ø	Ø	Ø	Ø	↓ (murine)	↓	↓ (murine)
Proliferation	↓/↑/↔↔↔	↑ (murine)	Ø	Ø	Ø	Ø	Ø	↓ (murine)
Expression of granule components								
Perforin	↓/↑↑/↔↔	Ø	Ø	Ø	↓↓	Ø	Ø	Ø
Granzymes	↑/↔↔	Ø	Ø	Ø	↓↓	Ø	Ø	↓ (murine)
Cytokine secretion								
IFN-γ	↓↓/↑↑/↔	Ø	↓	↓ (murine)	↓/↑	Ø	Ø	↓/↑ (murine)
TNF-α	Ø	Ø	Ø	Ø	Ø	Ø	Ø	↔ (murine)
IL-17	Ø	Ø	Ø	Ø	Ø	↑ (murine)	Ø	Ø
Maturation	Ø	Ø	Ø	↑ (murine)	Ø	Ø	Ø	Ø
Metabolic activity	↑	Ø	Ø	Ø	Ø	Ø	↓	Ø

*IFN-γ, interferon γ; NK cell, natural killer cell; TNF-α, tumor necrosis factor α; ↔, no change; ↑, increase; ↓, decrease; Ø, no data. Numbers of the arrow show the number of references*.

### Insights to Adiponectin

Aside from leptin, adiponectin is also a well-characterized adipokine. In contrast to leptin, plasma levels of adiponectin are reduced in obese compared to normal-weight individuals (102). Only a few studies exist investigating the influence of adiponectin on NK cell physiology. Data revealed evidence for the expression of the adiponectin receptors 1 and 2 (AdipoR1 and 2) on murine and human NK cells with expression levels of 15–90%, suggesting physiological effects of this adipokine on these immune cells (70, 78–81) (Table 1). The predominant expression of adiponectin receptors on the CD56^dim^CD16^bright^ NK cells and lower expression rate in CD56^bright^CD16^dim/neg^ NK cells indicate that adiponectin primarily influences the cytotoxic NK cell subset (79). Several results were found concerning the impact of adiponectin on NK cell function. Adiponectin incubation of IL-2-stimulated murine NK cells isolated from splenic tissue resulted in a reduced cytotoxicity, IFN-γ secretion, and expression of FasL and TRAIL (81, 103). Furthermore, adiponectin knockout mice exhibit a decreased CD107a and NKG2D receptor expression as well as a reduced frequency of CD11b^high^CD27^high^ NK cell subset—the most potent NK cell subset in mice—compared to wild-type mice (79). Accordingly, results of studies on human NK cells demonstrated that adiponectin was shown to increase CD107a expression and cytotoxicity of NK cells isolated from normal-weight humans (99), indicating a benefit of this adipokine on NK cell function. In line with the findings on leptin, adiponectin failed to enhance NK cell activity in obese humans (99). Moreover, studies on obese individuals showed that the expression of the adiponectin receptor 2 is reduced and that low plasma adiponectin concentrations correlated with a diminished cytolytic activity of NK cells from obese individuals (70, 81).

Taken together, data on adiponectin effects on NK cells are conflicting. Therefore, future studies on normal-weight compared to obese individuals could help to clarify these discrepancies.

### Effects of Interleukin-6

Interleukin-6 plasma concentrations are typically increased in obese individuals (104). This cytokine is one of the major inflammatory mediators in obesity. In addition to effects on macrophage polarization, IL-6 was demonstrated to influence NK cells directly (105). IL-6 receptor (IL-6R) expression was detected in murine blood, hepatic, adipose tissue, and splenic NK cells as well as in the human NK-92 cell line and human blood, splenic, and tonsil NK cells (82–86) (Table 1). Interestingly, the expression of the IL-6 receptor was increased in obese mice compared to normal-weight mice in blood and hepatic NK cells (84). In addition, a distinct IL-6 receptor-positive NK cell population was described to be a specific mediator of metaflammation (metabolic inflammatory state) and insulin resistance in obesity. In detail, an IL-6/Stat3-dependent formation on these NK cells seems to account to obesity-associated pathologies (84). Several investigations on NK cell physiology demonstrated a decreased cytotoxicity and IFN-γ secretion as well as perforin and granzyme expression of human NK cells induced by IL-6 treatment (106–110). However, conflicting results exist with an IL-6-mediated increase of cytotoxicity, cytokine secretion, and expression of activating receptors of NK cells in human and mice (85, 106, 111–114). In addition, expression levels of inhibiting or activating NK cell receptors were observed to be unchanged by IL-6 treatment (106). Of note, physical activity suppresses subcutaneous B16 melanoma tumor growth through epinephrine- and IL-6-dependent NK cell mobilization and redistribution in mice, suggesting a rather activating effect of IL-6 on NK cells (86). Future studies are necessary to evaluate possible dose-dependent differences as well as effects of IL-6 administration *in vivo* in lean and obese subjects to specify the meaning of an obesity-induced increase of IL-6 concentration on NK cell physiology.

### Studies on Estrogens

In addition to other sex steroid hormones, high BMI is strongly associated with increased estrogen levels, especially in postmenopausal women (115). This is at least in part caused by the obesity-associated adipose tissue inflammation that leads to a stimulation of aromatase activity—the key enzyme of estrogen biosynthesis.

Estrogen receptors (ESR) have been detected in murine uterine and splenic NK cells as well as in human peripheral blood NK cells (87–89) (Table 1). Strikingly and in contrast to other adipokines, research outcomes on murine and human NK cells all point to an inhibiting effect of estrogen on NK cell functionality. Thus, estrogens reduced the cytotoxicity, expression of activating receptors, migration, proliferation, metabolic activity, granzyme expression, and IFN-γ secretion of NK cells, whereas estrogens increased the expression of the inhibiting receptor CD94 (88, 116–124). Only one publication demonstrated an increase of IFN-γ protein levels on NK cells after estrogen treatment, whereby in the same study, gene expression of IFN-γ was reduced by estrogens (117). As most of the investigations have been performed on mice, future studies on estrogen receptor expression as well as estrogen effects on human NK cells with respect to obesity are desirable. Estrogens are widely used for hormone replacement or fertility therapy. As elevated circulating estrogen levels are highly associated with increased risk of breast cancer in obese postmenopausal women, more detailed studies are urgently needed to elucidate the role of estrogens on NK cells. Furthermore, cancer-protecting effects of the hormone would be of high clinical interest (125).

Insights about expression of adipokine receptors on NK cells as well as obesity-associated changes in receptor expression levels are presented in Table 1. Data about the influence of leptin, adiponectin, IL-6, and estrogen on number, functionality, receptor expression, and proliferation of NK cells are summarized in Table 2.

Besides investigations about the effect of single adipokines, some studies investigated the effect of an incubation of NK cells with an adipocyte-conditioned media (ACM) to simulate a physiologically mixture of components secreted by adipocytes. Results demonstrated a reduced cytotoxicity of ACM-treated human NK cells against prostate cancer cells. This effect has been shown to be primarily mediated via the IL-6 and leptin content in the ACM (126). Furthermore, ACM-treatment of NK cells resulted in a decrease of granzyme- and TRAIL-positive NK cells, but an increase of IFN-γ production, although the effects of ACM seem to be species-specific and depending on the different phases of adipogenesis the ACM was harvested (69, 77).

In summary, although many conflicting data were gained in the past, obesity-related changes of adipokine concentrations leading to an altered NK cell physiology are still obvious (Tables 1, 2). For certain, more data are needed to strengthen particular findings and studies focusing on effects of other specific adipokines. Experiments using a mixture of obesity-related metabolites on NK cells, more resembling the *in vivo* situation, would help to elucidate their role on NK cell activity.

## THE Impact of Systemic Obesity on NK Cells in Animal Studies

Several studies previously investigated the impact of obesity on NK cells (Table 3). Data exist about differences in NK cell numbers in peripheral blood and organs, although the results are conflicting. Numerous studies demonstrated a decrease of the NK cell amount in blood as well as in spleen, lung, and liver of obese rodents (93, 98, 131, 140–143). In contrast, other reports exist showing either no changes or an increase of the NK cell number in blood and different tissues of obese individuals (75, 93, 97, 131, 132, 136, 138, 142). These discrepancies may be explained by species- or strain-dependent metabolic characteristics or by differences in developmental, degradation, or migration status of NK cells. In addition, compartment-specific NK cell distribution had been described, with an increased NK cell number in blood and spleen, but a decreased amount in the liver tissue of obese rats compared to their lean littermates (142). Moreover, heterogeneous methods of measurement may have led to different results. The amount of NK cells in blood or tissue is usually specified as “total NK cell number” or “NK cell frequency as percentage of leukocytes or PBMCs.” As obesity is also known to be associated with alterations in the number of other blood cell types, obesity-related changes in NK cell frequency may additionally be a result of a shift of numbers of other immune cell types (145). Therefore, the indication of absolute NK cell numbers would help to make data obtained in different studies and under different conditions comparable. To our knowledge, only two studies regarding the proportion of CD11b/CD27 NK cell subsets in obese rodents exist until now. One study indicated no differences in blood, liver, and perigonadal adipose tissue in obese mice compared to lean animals (84). However, another showed an increase of the CD11b^+^/CD27^−^ subpopulation and a decrease of the CD11b^−^/CD27^+^ subpopulation in visceral adipose tissue (135) (Table 3).

**Table 3:**
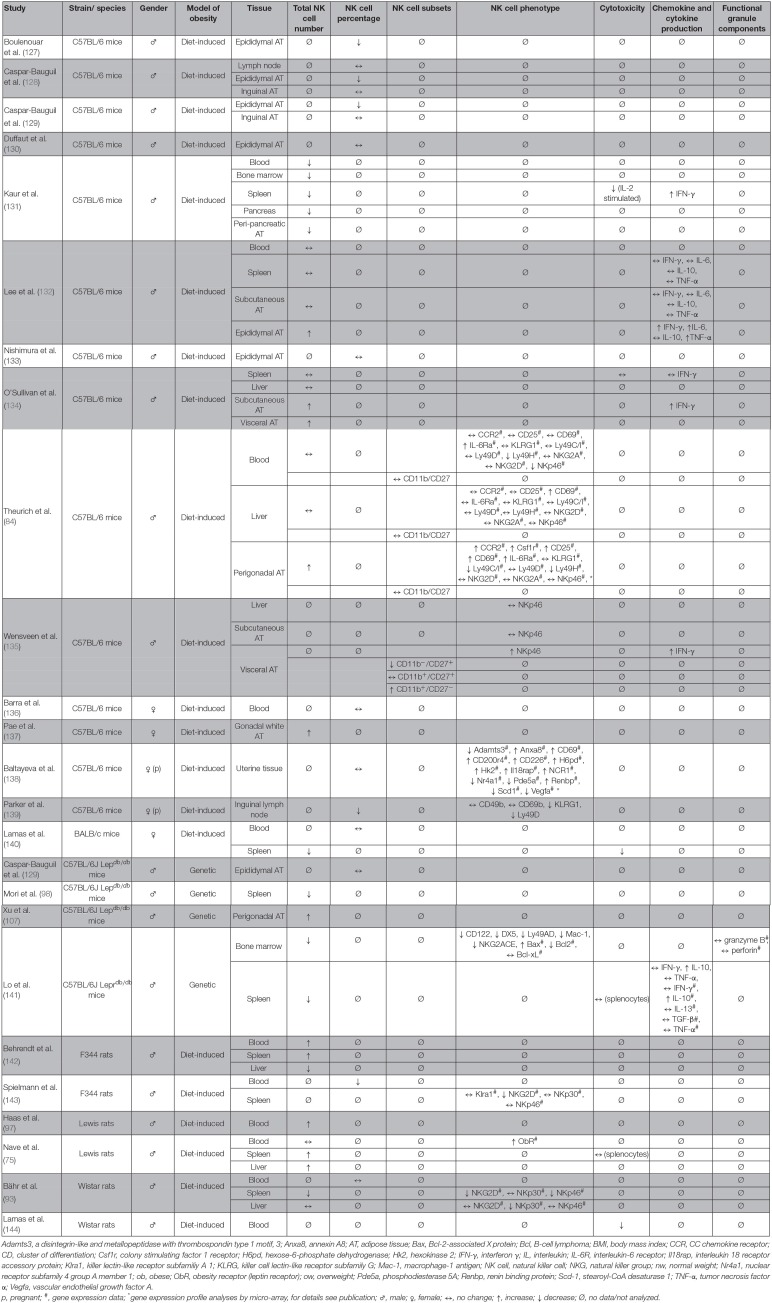
Overview of animal studies investigating the effect of obesity on number, subsets, phenotype, and functional parameters of NK cells in different tissues.

Adamts3, a disintegrin-like and metallopeptidase with thrombospondin type 1 motif, 3; Anxa8, annexin A8; AT, adipose tissue; Bax, Bcl-2-associated X protein; Bcl, B-cell lymphoma; BMI, body mass index; CCR, CC chemokine receptor; CD, cluster of differentiation; Csf1r, colony stimulating factor 1 receptor; H6pd, hexose-6-phosphate dehydrogenase; Hk2, hexokinase 2; IFN-γ, interferon γ; IL, interleukin; IL-6R, interleukin-6 receptor; Il18rap, interleukin 18 receptor accessory protein; Klra1, killer lectin-like receptor subfamiliy A 1; KLRG, killer cell lectin-like receptor subfamily G; Mac-1, macrophage-1 antigen; NK cell, natural killer cell; NKG, natural killer group; nw, normal weight; Nr4a1, nuclear receptor subfamily 4 group A member 1; ob, obese; ObR, obesity receptor (leptin receptor); ow, overweight; Pde5a, phosphodiesterase 5A; Renbp, renin binding protein; Scd-1, stearoyl-CoA desaturase 1; TNF-α, tumor necrosis factor α; Vegfa, vascular endothelial growth factor A.

p, pregnant;

^#^, gene expression data;

^*^ gene expression profile analyses by micro-array, for details see publication; ♂, male; ♀, female; ↔, no change; ↑, increase; ↓ decrease; Ø, no data/not analyzed.

However, in addition to changes in NK cell number, the expression of the activating NK cell receptors NKp46 and NKG2D in splenic tissue and NKp30 in liver tissue was reduced in diet-induced obese rats, indicating an inhibited activation status of NK cells in obesity (93, 143). Moreover, NK cells of obese rodents exhibit a reduced cytotoxicity against tumor cells compared to NK cells isolated from normal weight animals (13, 131, 140, 144). Interestingly, a study using adoptive transfer of NK cells demonstrated that NK cell functionality is dependent on the surrounding metabolic environment as the impaired phenotype of NK cells from obese rats could be ameliorated by transfer of NK cells into normal weight rats. These data implicated for the first time that the altered NK cell phenotype in obesity can be improved by generating the physiological metabolic environment of normal weight individuals (76).

## Effects of Systemic Obesity on Human NK Cells

In accordance to the results in mice and rats, obesity was shown to be associated with a decreased NK cell number in blood, liver, uterine, or colon tissue in humans (74, 99, 146–149), whereas several other studies did not observe any differences in the NK cell number comparing normal-weight and obese humans (71, 72, 84, 150–155) (Table 4). These discrepancies may be caused by the use of different markers and methods to identify NK cells and subsets in blood and different tissues and individual parameters in the study population, like gender, BMI, ethnicity, and nutritional or metabolic variances. Regarding functional parameters, obesity is associated with various alterations in NK cell phenotype. Data demonstrated that the expression of the functional marker TRAIL and the activating NK cell receptor NKp46 is reduced in obese individuals (72, 157). In contrast, studies reported a highly activated status of NK cells from obese humans with an increase of CD69, NKp46, and programmed cell death protein (PD)-1 expression as well as a decrease of the inhibiting NKG2A/CD94 complex and CD16 expression, which is known to be downregulated in activated NK cells (147, 157, 161). Despite the increased activation status, NK cells showed a decreased secretion of the cytokines granzyme B and perforin as well as the macrophage inflammatory protein (MIP)-1β after challenge with blood cancer cell lines in obese humans (147, 157). Furthermore, the degranulation capacity as well as the cytotoxicity against malignant cells is significantly impaired in obesity (99, 147, 157, 160). It has been discussed that the highly activated NK cells of obese individuals become exhausted much faster when exposed to target cells compared to NK cells of normal weight subjects, leading to an impaired capability to defend tumor or virus-infected cells (147, 157).

**Table 4:**
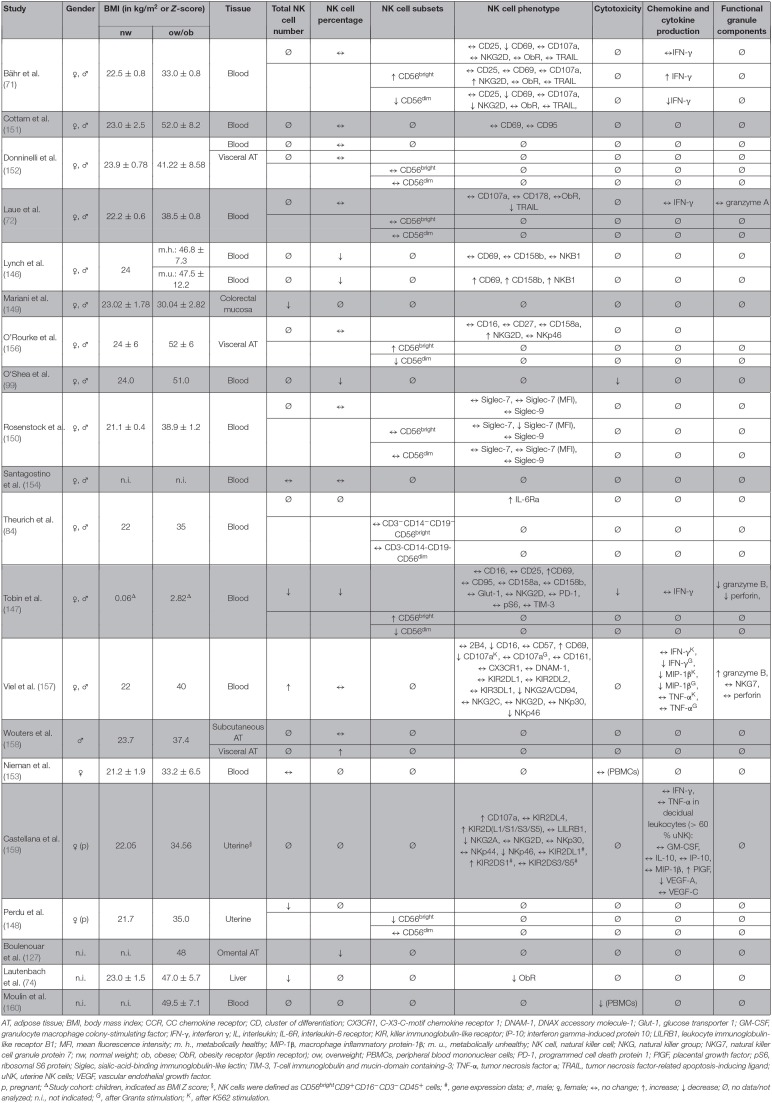
Overview of human studies investigating the effect of obesity on number, subsets, phenotype, and functional parameters of NK cells in different tissues.

AT, adipose tissue; BMI, body mass index; CCR, CC chemokine receptor; CD, cluster of differentiation; CX3CR1, C-X3-C-motif chemokine receptor 1; DNAM-1, DNAX accessory molecule-1; Glut-1, glucose transporter 1; GM-CSF, granulocyte macrophage colony-stimulating factor; IFN-γ, interferon γ; IL, interleukin; IL-6R, interleukin-6 receptor; KIR, killer immunoglobulin-like receptor; IP-10; interferon gamma-induced protein 10; LILRB1, leukocyte immunoglobulin-like receptor B1; MFI, mean fluorescence intensity; m. h., metabolically healthy; MIP-1β, macrophage inflammatory protein-1β; m. u., metabolically unhealthy; NK cell, natural killer cell; NKG, natural killer group; NKG7, natural killer cell granule protein 7; nw, normal weight; ob, obese; ObR, obesity receptor (leptin receptor); ow, overweight; PBMCs, peripheral blood mononuclear cells; PD-1, programmed cell death protein 1; PlGF, placental growth factor; pS6, ribosomal S6 protein; Siglec, sialic-acid-binding immunoglobulin-like lectin; TIM-3, T-cell immunoglobulin and mucin-domain containing-3; TNF-α, tumor necrosis factor α; TRAIL, tumor necrosis factor-related apoptosis-inducing ligand; uNK, uterine NK cells; VEGF, vascular endothelial growth factor.

p, pregnant;

*^Δ^Study cohort: children, indicated as BMI Z score; ^§^, NK cells were defined as CD56^bright^CD9^+^CD16^−^CD3^−^CD45^+^ cells; ^#^, gene expression data; ♂, male; ♀, female; ↔, no change; ↑, increase; ↓ decrease; Ø, no data/not analyzed; n.i., not indicated; ^G^, after Granta stimulation; ^K^, after K562 stimulation*.

Aside from the characterization of total NK cells, recent studies investigated the impact of obesity on the CD56^dim^CD16^bright^ and the CD56^bright^CD16^dim/neg^ NK cell subsets. Whereas, several studies did not find any differences on the CD56^dim^/CD56^bright^ ratio comparing NK cells isolated from normal-weight and obese humans (72, 150, 152, 156), results of two studies revealed evidence for a decrease of CD56^dim^CD16^bright^ NK cells and an increase of CD56^bright^CD16^dim/neg^ NK cells in obesity (71, 147). As previous studies observed that the CD56^dim^CD16^bright^ NK cell subset can convert into the CD56^bright^CD16^dim^ subset under the influence of specific cytokines and hormones, it had been assumed that obesity-related metabolites induce a conversion of CD56^dim^CD16^bright^ in CD56^bright^CD16^dim/neg^ NK cells in obese individuals (71, 162–164). Analyses of the subset-specific NK cell phenotype also demonstrated a decrease in NKG2D-positive and CD69-positive CD56^dim^CD16^bright^ NK cells (71). In addition, data on NK cell cytokine production demonstrated a decreased expression of IFN-γ-positive CD56^dim^CD16^bright^ NK cells in obese subjects, indicating an downregulation of the immunoregulatory effect of this NK cell subset in obesity (71). The lower CD56^dim^CD16^bright^ /CD56^bright^ CD16^dim/neg^ ratio as well as the reduced expression the activation marker CD69 and the activating receptor in the cytotoxic CD56^dim^CD16^bright^ NK cell subset under obese conditions may be one cause for the impaired cytotoxicity of NK cells against virus-infected and tumor cells and therefore contributes to the higher cancer risk and susceptibility for infections in obese subjects.

Investigations on children have revealed an impaired NK cell physiology even in childhood obesity. Obese children had a reduced NK cell frequency, which negatively correlated with BMI and insulin resistance. In addition, NK cells of obese children were highly activated and metabolically stressed but had significantly lower capabilities for target cell lyses and cytokine secretion compared to NK cells of normal-weight children (147). These data provided the first evidence for obesity-mediated alterations in NK cell physiology in childhood obesity and that this may contribute to the increased risk of obese children for developing infections and cancer diseases in adulthood.

Interestingly, some studies indicated that some of the impaired functional parameters are normalized in ex-obese individuals, as the CD69 expression and granzyme B secretion was decreased and IFN-γ production was increased after the loss of body weight and fat mass in obese adults (73, 157). In accordance with results of animal studies, these data indicate that the altered NK cell phenotype is dependent on the metabolic environment and can be reactivated by weight loss also in humans.

As obesity gets more and more in scientific and clinical focus, a subgroup of metabolically healthy obese individuals have been classified and is constantly more precisely defined (165, 166). Lynch et al. demonstrated that obese patients with an unhealthy metabolic profile exhibit less circulating NK cells as well as an altered NK cell phenotype compared to metabolically healthy patients. In detail, metabolically unhealthy obese patients had an increased expression of the activation marker CD69 and the inhibition markers NKB1 and CD158b on NK cells, compared to healthy obese individuals (146). This suggests that being unhealthy obese contributes to obesity-related complications and diseases through the negative influence on immune cells.

Moreover, in recent years, a group of so-called “normal-weight obese” individuals has been distinguished. This group is defined by a normal body weight and BMI, but an elevated body fat percentage, comparable to that of obese individuals. There are already studies showing similar associations of normal weight obesity with metabolic syndrome and insulin resistance and obesity related co-morbidities (167–169). Less is known about the impact of normal weight obesity on immune cells and the resulting risk for cancer burden.

## Tissue-Resident NK Cells in Adipose Tissue of Obese Individuals

Obesity is not only accompanied by an adipocyte hypertrophy and hyperplasia but also with an increase of infiltration of immune cells. Numbers of macrophages, T cells, and NK cells increase in adipose tissue and therefore indicate a chronic low-grade inflammation of the fat tissue itself and also systemically. Thus, in recent years, the characterization of NK cells in distinct adipose tissue depots became of scientific interest. Generally, NK cells represent about 13% of all leukocytes in visceral adipose tissue (135). Characterization of NK cell subpopulations in the stromal vascular fraction of human visceral adipose tissue of lean healthy subjects demonstrated an accumulation of CD56^bright^CD16^dim/neg^ NK cells (about 30% of total NK cells, compared to 10% in peripheral blood) and a lower frequency of CD56^dim^CD16^bright^ NK cells (about 70% of total NK cells, compared to 90% in peripheral blood) compared to the NK cell subset distribution in the peripheral blood (152).

Several studies reported an increase of the NK cell number in the adipose tissue of leptin-deficient and diet-induced obese mice as well as in the adipose tissue of obese humans (84, 107, 132, 134, 135, 137, 158). This elevated NK cell number is primarily induced by an increased proliferation of tissue-resident NK cells and to a lesser extent by recruitment from the periphery (134, 135). Interestingly, data indicate that the obesity-associated change in NK cell numbers especially occurs in the metabolically active visceral adipose tissue, but not in subcutaneous adipose tissue (132, 135, 158). Nevertheless, in contrast, few studies also demonstrate a decrease of NK cell numbers in adipose tissue of obese mice or humans or could not observe any differences in NK cell numbers comparing adipose tissue of normal weight and obese individuals (128–130, 133). These discrepancies may result from different feeding periods or composition of high-fat diets, definition of NK cells in flow cytometry analyses, or the inconsistent measurement of total NK cell number or NK cell frequency.

Analyzing the functionality of adipose tissue-resident NK cells, animal studies demonstrated an increased secretion of IL-6, IFN-γ, and TNF-α by NK cells isolated from visceral and subcutaneous adipose tissue of obese mice (132, 134, 135). In subcutaneous adipose tissue of obese individuals, a shift from the cytotoxic CD56^dim^ NK cell subset to the cytokine secreting CD56^bright^ NK cell subpopulation could be observed (155). This might be one explanation for the increased cytokine secretion of adipose tissue NK cells in obesity. Interestingly, analyses of the stromal vascular fraction of omental adipose tissue from obese and ex-obese patients showed a vanished CD56^bright^ subset and, in contrast to peripheral blood, an increase of the CD16 expression within the CD56^dim^ subset (127). Moreover, obesity drove an upregulation of ligands of the activating receptor NKp46 on adipocytes of obese mice, which stimulated the proliferation and IFN-γ secretion of NK cells and, consequently, the polarization from the anti-inflammatory M2 macrophages to the pro-inflammatory M1 macrophages (135). In addition, systemic NK cell ablation in mice decreased the infiltration of macrophages in visceral adipose tissue (156). Therefore, NK cells have been discussed to contribute to the adipose tissue inflammation including the polarization of pro-inflammatory macrophages in obesity (132, 134, 135). Interestingly, a reduction of body weight and body fat mass by caloric restriction was associated with a decreased NK cell number in inguinal adipose tissue of diet-induced obese mice, leading to the assumption that weight loss may prevent adipose tissue inflammation in obese individuals (170).

Investigating the expression of activating NK cell receptors, studies on human subcutaneous adipose tissue NK cells demonstrated that the expression of the NKp30 and NKp44 receptors were decreased in obese subjects, whereas the NKG2D expression levels were not affected by obesity (155). These data indicate a reduced cytolytic activity of NK cells in subcutaneous adipose tissue of obese subjects, which might contribute to the higher susceptibility to infections and increased cancer risk under obese conditions. Future investigations focusing on the cytotoxic activity of adipose tissue NK cells of normal weight and obese individuals against tumor cells are necessary to complement the knowledge about the impact of NK cells in different adipose tissues. Moreover, the effect of body weight and fat mass reduction as well as physical activity on adipose tissue NK cells and inflammatory parameters would be of high interest.

## The Link Between NK Cells and the Development of Obesity-Associated Inflammation and Insulin-Resistance

Changes in NK cell phenotype has also been shown in obese patients with comorbidities, especially type 2 diabetic patients. Romero et al. demonstrated a reduced frequency of the activating NK cell receptor KIR2DS4 in obese diabetic patients compared to normal-weight diabetic patients (171). Moreover, NK cells of obese patients with new onset type 2 diabetes mellitus exhibited an increase of NKG2D expression and degranulation capacity as well as a reduced NKG2A and KIR2DL3 expression in peripheral blood compared to normal-weight control patients (172). Besides the assumption that metabolic changes, like chronic low-grade inflammation, in obesity lead to an impaired NK cell physiology, recent studies demonstrated on the other hand that an altered NK cell function may contribute to the development of inflammatory processes and disturbances of glucose metabolism. Mice fed a high-fat diet revealed a significantly higher accumulation of pro-inflammatory M1 macrophages in adipose tissue compared to normal-weight mice, which was prevented by NK cell depletion in these animals (127, 132, 135). These results indicate that NK cells induce adipose tissue inflammation by enhancing the macrophage polarization, which was mediated via upregulation of NKp46 ligands on adipocytes and, consequently, a stimulation of IFN-γ secretion by NK cells (132, 134, 135). In addition, results of these studies reported that NK cell-depleted mice as well as mice lacking the NKp46 receptor fed with a high-fat diet failed to develop hyperinsulinemia and exhibited an improved glucose tolerance and insulin sensitivity (132, 134, 135). Investigations of Theurich et al. revealed that obesity is associated with an increase of an IL-6/Stat3-dependent expansion of a distinct IL-6Ra- and Csf1r (colony stimulating factor 1 receptor)-expressing NK cell subpopulation in mice and humans, which are important mediators of metaflammation and insulin resistance (84).

In summary, these data suggest that alterations in NK cell physiology may also contribute to obesity-associated pathogenesis of insulin resistance and type 2 diabetes mellitus.

## The Impact of Obesity-Related Altered NK Cell Functionality in the Defense of Infection and Tumor Development

Little information exists about the influence of obesity-associated disturbances of NK cell physiology on the high susceptibility for infections and increased cancer risk in obese individuals.

So far, only limited studies investigated the impact of obesity on NK cell functionality during infections. An animal study from Smith et al. demonstrated a reduced cytotoxicity of NK cells isolated from obese mice infected with influenza virus, combined by an increased mortality rate compared to normal-weight animals with influenza infection (13). In humans, a relation of the high incidence of H1N1 infections and disturbed NK cell functions in obese individuals is proposed (173). Latest data also provided evidence for a decreased NK cell number in mice orally infected with *Salmonella* Typhimurium, which also may contribute to an impaired susceptibility for infections in obese individuals (174). Interestingly, it has been proposed that selective therapeutic drugs might get trapped in adipose tissue in the treatment of HIV infections and, furthermore, immune cells, including NK cells, could be retained in adipose tissue, blocking their immunosurveillance elsewhere in the body (175).

Several studies addressed obesity-related alterations in NK cell functionality during tumor development. Animal studies on diet-induced rats injected with a metastatic breast cancer cell line reported a reduced NK cell number in the blood as well as a decrease of NK cell-tumor cell interactions in the lung, compared to lean animals. Furthermore, obese rats showed reduced NKG2D expression levels in the spleen combined with an enhanced number of lung metastasis (143). Accordingly, obese mice injected with breast cancer cells revealed a lower NK cell number in blood and spleen as well as a reduced NK cell cytotoxicity against lymphoma cells, compared to their lean littermates (140).

In addition to investigations on breast cancer, several lines of evidence show an impact of altered NK cell functionality in obese individuals and colon cancer development. Studies in rats with chemically induced colon cancer demonstrated a decreased number of NK cells in liver, spleen, and colon tumors as well as reduced expression levels of activating NK cell receptors in splenic tissue (NKG2D and NKp46) and liver tissue (NKp30) of diet-induced obese animals. This was associated with an increased quantity, size, and weight of colon tumors in obese rats, compared to normal-weight animals (93). In accordance, results of human studies reported a reduced NK cell number in normal colon tissue of obese patients compared to normal-weight patients, which may contribute to an increased risk for the development of colon cancer in obesity (149). Moreover, analyses of human NKG2D genotypes indicated that subjects with a NKG2D genotype associated with a high NK cell activity exhibit a decreased risk to develop colon cancer (176). In contrast, no differences in the number or subset distribution of NK cells had been observed in blood or adipose tissue of patients with colorectal cancer (152).

Besides investigations in the context of breast and colon cancer, animal studies on leptin-deficient obese (*ob/ob*) mice injected with melanoma cells showed a significant decreased splenic NK cell number in obese mice compared to normal weight wild-type control mice. Interestingly, this effect was reversible by leptin administration (98). A recent study demonstrated associations between obesity-related alterations in NK cell physiology and pancreatic cancer development. Obese mice with KRAS mutation had a decreased NK cell number in blood, spleen, and bone marrow as well as a reduced NK cell cytotoxicity, compared to their normal weight littermates at the pre-malignant stage of pancreatic tumorigenesis. This has been discussed to contribute to the enhanced progression of cancer from a dysplastic stage to invasive cancer in obese individuals (131).

Additionally, the migration of NK cells under obese conditions was in the focus. Among leukocytes, NK cells are the most active migrating cells (177). A study from our group investigated the time-dependent influence of different leptin concentrations on the cellular morphology of NK-92 cells (91). The study revealed an impact of physiological concentrations of leptin on the filopodia length and of the co-localization of cofilin and F-actin, suggesting an increased motility of NK cells and a possible support of immune defense against tumor cells.

The latest studies have demonstrated that obesity is associated with a peroxisome proliferator-activated receptor (PPAR)-driven lipid accumulation in NK cells, leading to metabolic dysfunctions, like inhibition of perforin, IFN-γ, and granzyme B secretion as well as target cell lysis (178). These lipid-induced metabolic defects of NK cells were shown to be associated with a loss of antitumor response (178).

**Figure 1:**
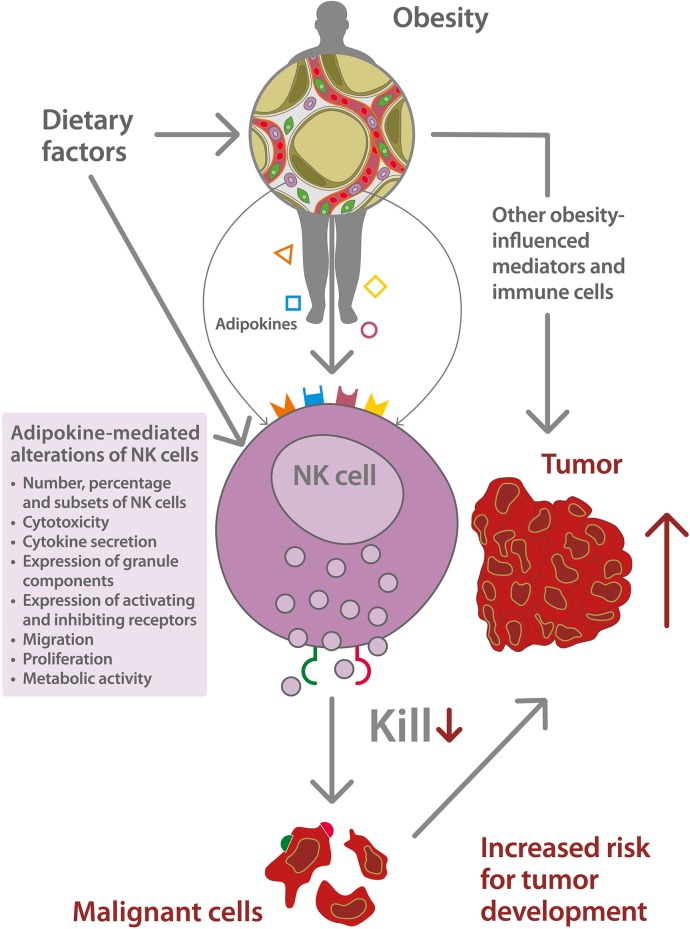
Schematic depiction of the impact of natural killer (NK) cell dysfunction on the increased risk for tumor development in obesity. Dietary factors, like high energy intake or high intake of saturated fatty acids, promote the development of overweight and obesity and mediate direct effects on NK cell function. Obesity is associated with an excessive growth of adipose tissue and a dysregulated secretion of adipokines, like leptin (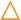), adiponectin (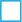), interleukin-6 (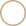), estrogens (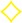), and others. The obesity-related metabolic environment leads to an altered NK cell functionality including a dysbalance of the expression of activating and inhibiting NK cell receptors as well as NK cell receptor ligands (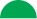, 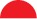) on tumor cells. This NK cell dysfunction is associated with a decreased killing capacity of malignant cells and an increased risk for tumor development.

A schematic depiction of the relationship between obesity-associated alterations of NK cells, and the increased risk for tumor development is displayed in Figure 1.

### NK Cell-Based Immunotherapies in Cancer Treatment

A large number of scientific investigations and clinical studies have demonstrated that NK cells have a great potential for cancer immunotherapy (179). Besides the application of NK cell stimulating cytokines, the treatment of cancer patients with antibodies targeting inhibitory NK cell receptors or agonists of activating NK cell receptors were used to increase the number and functionality of NK cells. Very recently, a review by Nersesian et al. demonstrated the success of NK cell-based therapy in patients with ovarian cancer; however, aspects of obesity were not touched (180). In addition, NK cell adoptive transfer therapies for cancer patients using allogenic blood NK cells, stem cell-derived NK cells, or different NK cell lines exhibited promising effects in the treatment of several cancer types, whereas only limited clinical benefits were observed in cancer patients who received an adoptive transfer of autologous NK cells (181–183). Moreover, the use of genetically modified NK cells that express a chimeric antigen receptor (CAR) led to an optimized therapeutic potential of adoptive NK cell transfer therapies (184). Despite the large number of pre-clinical and clinical studies on NK cell-based immunotherapies, no data exist addressing a possible influence on therapeutic effects in the case of obese NK cell donors or obese recipients. With the knowledge from animal studies that an obese metabolic environment alters NK cell functionality and phenotype, an impaired therapeutic effect of adoptive NK cell transfer therapies in obese patients or the use of NK cells isolated from obese individuals for immunotherapies might be assumed (76).

## Reactivation of NK Cells by Body Weight Reduction and Physical Activity

In addition to findings on altered NK cell physiology in obesity, a number of studies exist investigating obesity-preventing aspects, like body weight reduction by caloric restriction or bariatric surgery as well as physical activity on NK cells.

Investigations analyzing the effects of caloric restriction on NK cells demonstrated no changes or a decreased NK cell number after the dietary intervention in obese and non-obese mice and humans (170, 185–187). However, NK cell cytotoxicity in obese rats and humans as well as in non-obese humans has been shown to be increased after energy restriction or low-fat diet (144, 188, 189). In addition, caloric restriction was associated with a higher expression of the activation marker CD69 and B220 as well as an increased secretion of TNF-α and GM-CSF in non-obese mice (185). Although the majority of data indicate a stimulating effect of caloric restriction on NK cell functionality, a few studies reported contrary results with a reduced killing activity, an impaired maturation, and IFN-γ production of NK cells after caloric restriction in non-obese mice or obese woman (185, 187). Interestingly, life-long weight stability and prevention of weight cycling has been shown to be associated with a higher lytic activity of NK cells against tumor cells, providing evidence that frequent intentional weight loss may negatively influence NK cell function (190).

Aside from energy restriction via conventional dietary intervention, the loss of body weight and fat mass 6 months after bariatric surgery in obese patients clearly increased the cytotoxicity and cytokine production of NK cells (160). In contrast, another study demonstrated a reduced expression of the activation marker CD69 and the Fas antigen CD95 in obese patients 12 months after gastric bypass surgery (151). These contrary findings may result from different surgery methods, the intensity of weight loss, or the post-operative time the samples were analyzed.

Physical activity is one of the preventive strategies to inhibit the development of overweight and obesity and is the most effective intervention to reduce body weight and fat mass as well as to decrease the risk for secondary diseases in obese individuals. In the last 30 years, numerous studies demonstrated an increased number and cytotoxicity of NK cells during and after a moderate and intensive training program in mice and humans (86, 136, 170, 191–202). These effects seem to be independent of the type of the physical activity and have been shown for walking, running, marathon, race cycling, or aerobics. In addition, in these studies, activation of NK cells has been demonstrated by acute training programs as well as by experimental settings with chronic exercise. Most of the studies initially reported an acute stimulating effect of physical activity on NK cells during or right after the training session, followed by a recurrence back to basal levels after minutes or few hours (191–193, 197, 203). The increase of the NK cell numbers under exercise conditions has been proposed to be induced by a rapid mobilization of tissue-resident NK cells into the circulation, possibly caused by the increase of released cytokines, like IL-6, IL-7, or IL-15, from muscle cells (204–208). In addition to NK cell number and cytotoxicity, NK cell subset distribution is affected by exercise. Whereas, two studies demonstrated an increase in the CD56^dim^CD16^bright^ NK cell subset, other studies demonstrated a higher frequency of the CD56^bright^CD16^dim/neg^ NK cell subpopulation (86, 203, 209, 210). However, physical activity increased the expression of the activating NK cell receptors CD69 and NKG2D and the degranulation marker CD107a as well as the IFN-γ, perforin, and granzyme B content of NK cells (73, 196, 203, 211). Furthermore, the expression of the inhibitory NK cell receptor KLRG1 was reduced after exercise training (203). Taken together, the data provide convincing evidence for a NK cell stimulating effect of physical activity in normal-weight and obese individuals.

Until now, only two studies exist investigating the impact of exercise on NK cell physiology and the association with tumor development. A murine experiment demonstrated an increased NK cell infiltration in subcutaneous adipose and lung tumor tissues associated with an inhibited tumor growth in mice after voluntary wheel-running (86). This NK cell infiltration in the tumor tissue was proposed to be induced by the enhanced IL-6 release after physical activity. Furthermore, exercise led to an NK cell-activating milieu in the tumor tissue by increasing the expression of NK cell-activating ligands as well as a more pronounced release of stimulatory cytokines and chemoattractant chemokines (86). In addition, high-intensity interval training led to an increased number and activation status of blood NK cells and a reduced lung tumor burden in obese mice (136). These findings suggest that the enhanced risk for tumor development in obesity might be reduced by an exercise-induced activation of NK cells.

## Conclusion and Outlook

In conclusion, the herein discussed data clearly demonstrate an association of impaired NK cell functionality with obesity. Among other factors, altered NK cell biology contributes to an increased cancer risk and a more severe cancer outcome in obese individuals (99). Future research is urgently needed to elucidate conflicting results in the field and to break down the most relevant alterations in NK cell functions. Obesity-related changes in concentrations and signaling cascades of adipokines might be primarily responsible for the alterations of NK cells. Nevertheless, in the future, other endocrine and metabolic factors associated with obesity and their influence on NK cells should be studied in more detail. Even though, available data are heterogeneous, obesity-related changes in activating NK cell receptors NKG2D and NKp46 were found in various studies. As obesity is one of the most important preventable risk factors for the development of cancer, more investigations on the potency of weight loss and physical activity as therapeutic interventions for cancer should be carried out. Pioneering work shows a positive feedback loop of physical activity and fat mass reduction on NK cell functionality that most likely control cancer development. Furthermore, NK cell therapies are promising components of modern immunotherapies to treat cancer. NK cell donor BMI will certainly have an impact on the success rate of the therapeutic intervention. Thus, studies elucidating the role of donor BMI on NK cell therapy outcome are of high interest.

NK cells are sensitive to obesity-related changes on multiple yet not fully elucidated levels and significantly shape infection and cancer outcome.

## Author Contributions

IB and JS took the lead in writing the manuscript. DQ and HK contributed to the writing of the manuscript and provided critical feedback for the manuscript. HK supervised the underlying projects of the working group.

### Conflict of Interest

The authors declare that the research was conducted in the absence of any commercial or financial relationships that could be construed as a potential conflict of interest.

## Abbreviations

ACM: adipocyte-conditioned media
Adamts3: a disintegrin-like and metallopeptidase with thrombospondin type 1 motif, 3
AdipoR: adiponectin receptor
Anxa8: annexin A8
AT: adipose tissue
Bax: Bcl-2-associated X protein
Bcl: B-cell lymphoma
BMI: body mass index
CCR: CC chemokine receptor
Csf1r: colony stimulating factor 1 receptor
CX3CR1: C-X3-C-motif chemokine receptor 1
DNAM-1: DNAX accessory molecule-1
ESR: estrogen receptor
FasL: Fas ligand
IFN-γ: interferon
GM-CSF: granulocyte macrophage colony-stimulating factor
H6pd: hexose-6-phosphate dehydrogenase
Hk2: hexokinase 2
IL: interleukinIL-6R; interleukin-6 receptor
Il18rap: interleukin 18 receptor accessory protein
IP-10: interferon gamma-induced protein 10
JAK: Janus kinase
KIR: killer immunoglobulin-like receptor
Klra1: killer lectin-like receptor subfamiliy A 1
KLRG: killer cell lectin-like receptor subfamily G
LEP-R: leptin receptor
LILRB1: leukocyte immunoglobulin-like receptor B1
Mac-1: macrophage-1 antigen
MFI: mean fluorescence intensity
m.h.: metabolically healthy
MIP-1β: macrophage inflammatory protein-1β
m. u.: metabolically unhealthy
NK cell: natural killer cell
NKG: natural killer group
NKG7: natural killer cell granule protein 7
Nr4a1: nuclear receptor subfamily 4 group A member 1
Nw: normal weight
Ob: obese
ObR: obesity receptor (leptin receptor)
Ow: overweight
PAQR: progestin and adipoQ receptor
PBMCs: peripheral blood mononuclear cells
PD-1: programmed cell death protein-1
Pde5a: phosphodiesterase 5A
PlGF: placental growth factor
PPAR: peroxisome proliferator-activated receptor
pS6: ribosomal S6 protein
Renbp: renin binding protein
Scd-1: stearoyl-CoA desaturase 1
Siglec: sialic-acid-binding immunoglobulin-like lectin
STAT: signal transducer and activator of transcription
TGF-β: transforming growth factor
TIM-3: T-cell immunoglobulin and mucin-domain containing-3
TNF-α: tumor necrosis factor
TRAIL: tumor necrosis factor-related apoptosis-inducing ligand
uNK: uterine NK cells
VEGF: vascular endothelial growth factor
Vegfa: vascular endothelial growth factor A.
